# Endothelial/Epithelial Mesenchymal Transition in Ascending Aortas of Patients With Bicuspid Aortic Valve

**DOI:** 10.3389/fcvm.2019.00182

**Published:** 2019-12-17

**Authors:** Shohreh Maleki, Flore-Anne Poujade, Otto Bergman, Jesper R. Gådin, Nancy Simon, Karin Lång, Anders Franco-Cereceda, Simon C. Body, Hanna M. Björck, Per Eriksson

**Affiliations:** ^1^Cardiovascular Medicine Unit, Center for Molecular Medicine, Department of Medicine, Karolinska Institutet, Karolinska University Hospital, Solna, Sweden; ^2^Cardiothoracic Surgery Unit, Department of Molecular Medicine and Surgery, Karolinska Institutet, Karolinska University Hospital, Solna, Sweden; ^3^Department of Anesthesiology, Perioperative, and Pain Medicine, Brigham and Women's Hospital, Harvard Medical School, Boston, MA, United States

**Keywords:** bicuspid aortc valve, aneurysm, endothelial to mesenchymal transition (EndMT), ascending aorta, endothelial cell (EC)

## Abstract

Thoracic aortic aneurysm (TAA) is the progressive enlargement of the aorta due to destructive changes in the connective tissue of the aortic wall. Aneurysm development is silent and often first manifested by the drastic events of aortic dissection or rupture. As yet, therapeutic agents that halt or reverse the process of aortic wall deterioration are absent, and the only available therapeutic recommendation is elective prophylactic surgical intervention. Being born with a bicuspid instead of the normal tricuspid aortic valve (TAV) is a major risk factor for developing aneurysm in the ascending aorta later in life. Although the pathophysiology of the increased aneurysm susceptibility is not known, recent studies are suggestive of a transformation of aortic endothelium into a more mesenchymal state i.e., an endothelial-to-mesenchymal transition in these individuals. This process involves the loss of endothelial cell features, resulting in junction instability and enhanced vascular permeability of the ascending aorta that may lay the ground for increased aneurysm susceptibility. This finding differentiates and further emphasizes the specific characteristics of aneurysm development in individuals with a bicuspid aortic valve (BAV). This review discusses the possibility of a developmental fate shared between the aortic endothelium and aortic valves. It further speculates about the impact of aortic endothelium phenotypic shift on aneurysm development in individuals with a BAV and revisits previous studies in the light of the new findings.

## Introduction

Thoracic aortic aneurysm (TAA) is a potentially deadly disease associated with progressive expansion and degeneration of the aorta. One of the highest risk factors for developing TAA is the possession of a bicuspid aortic valve (BAV) instead of the normal tricuspid aortic valve (TAV). BAV is the most common congenital cardiac disorder, more frequent in males and Caucasians and has a prevalence of 0.5–2% in the human population (1). Importantly, individuals with a BAV have an 80 times increased risk of developing aortic aneurysm compared to the general population (2). BAV is a complex disease with unknown etiology for the higher aneurysm susceptibility, and the importance of inheritance vs. exposure of ascending aortas (AscA) to non-physiological hemodynamics is currently debated.

The inheritance of BAV in human has been intensively studied and is beyond the scope of this review. Briefly, several genes i.e., *NOTCH1, ACTA2, GATA4/5, NKX2.5*, and *SMAD6* have been characterized in association with familial non-syndromic BAV (1, 3–5). As yet, the high prevalence of sporadic BAV is not compatible with the few characterized genes for familial inheritance and this area of research is still open for new findings. Regarding the influence of shear stress, the last decade has witnessed a major breakthrough in studying the non-physiological hemodynamics caused by a BAV and its possible impact on AscA pathogenesis. Numerous original research and review articles have been allocated to this subject to which the interested readers can refer (6–12). With increasing data obtained on non-physiological hemodynamic of BAV patients, the common consensus emerging is that both genetics and hemodynamics contribute to aortopathy in BAV.

We and others have shown that ascending aortic aneurysm has different etiologies in patients with BAV and TAV [e.g., (13, 14)]. A deeper insight into ongoing molecular processes in the AscA prior to and after aneurysm manifestation is a prerequisite for understanding and preventing aortic degeneration. Moreover, discovering the inheritance of BAV aorthopathy, i.e., the set of genetic and/or epigenetic alterations that leads to AscA aneurysm coupled to a BAV, requires detailed cellular and molecular knowledge of interactions between different embryonic progenitors that act at the common window of space and time to determine the fate of aortic valve and AscA simultaneously.

Two recently published articles by us and others, showed an alteration of intimal endothelium in aneurysmal (15) and non-aneurysmal (16) BAV AscA to a more mesenchymal phenotype and discussed the possible contribution of the phenomenon endothelial mesenchymal transition (EndMT) to the development of aneurysm in these patients. These, and a number of other relevant observations, open up a new avenue in the field of aneurysm. As is highlighted in the title, this review will concentrate only on possible mechanisms of induction and cellular/molecular impact of the EndMT process on the higher susceptibility to develop aneurysm in individuals with BAV. The second objective is to explore if induction of this process in the intima and, as we have observed and will discuss later in this review, most probably also in the media, would clarify better the differences in onset and extent of disease manifestation and pathological changes induced by aneurysms in AscAs of humans with a BAV. Hence, throughout this review we use the term EndMT/EMT (epithelial mesenchymal transition) to describe the result obtained from intima-media of AscA and EndMT when observation is limited to the endothelial layer. We hope this review will widen the scope and add new dimensions and perspectives to the field of aneurysm research.

## Embryonic Development of Heart: Relationships Between Aortic Valves and Ascending Aortas

To explore the possible connection between the formation of a BAV and altered endothelial function in AscA, we should first consider the developmental context within which the fate of semilunar valves (aortic and pulmonic valves) and ascending aortic endothelium is determined. This requires a short review of the cardiac development and formation of cardiac cushion or primordia of aortic valves from endocardium. In the coming sections, we summarize a set of experiments done in transgenic models that have aided us to gain a clearer picture of the inter-connection between embryogenesis of aortic valves and the AscA.

In the human embryo, the linear heart tube forms by differentiation of cardiomyocytes within the primitive cardiac mesoderm, termed the cardiac crescent, during the third week of embryonic development (17). The heart tube is composed of the inner lining/endocardium and an outer layer/myocardium, separated by extracellular matrix known as cardiac jelly. Later during gestation, the cardiac tube loops and elongates by the addition of myocardium and mesenchymal tissues lying outside the early heart; the second heart field (SHF) progenitors and migrating cardiac neural crest cells (NCC). During looping, the first manifestation of cardiac valve formation appears as the expansion and swelling of the cardiac jelly in the atrioventricular canal, and somewhat later in the outflow tract (OFT), form the cardiac cushions via EndMT. Development and remodeling of semilunar valves and OFT septation into the AscA and the pulmonary trunk is accomplished by concerted interaction of OFT cushion with NCC and SHF progenitors with the net effect of complete separation of the systemic and pulmonary circulation (14, 18–21).

The pioneering set of experiments performed by Jonathan Epstein's group (19, 22–24), provided a framework for explaining the relationship between the morphogenesis of aortic valves and AscA. According to this model, the development of the aortic valve and primitive AscA is spatiotemporally related and involves a coordinated signal exchange between endocardium, SHF and NCC progenitors. Impaired function of each cardiac progenitor influences the embryonic development of the other, as well as the endocardia EndMT/EMT and cushion formation, resulting in the formation of a BAV or other aortic valves anomalies. One example of such interaction was shown recently by formation of a BAV as a consequence of deletion of an endothelial specific gene *NOS3* in mice, manifesting impaired distribution of NCC and SHF within the aortic valve leaflets (21).

The model was further backed by experiments demonstrating that proper formation and/or septation of the OFT into AscA and pulmonary trunk was also tightly coupled to signals generated from endocardial cushion and cardiac progenitors and abolition of function in any of the involved compartment, SHF (25, 26), NCC (27–29), or endocardium (30, 31) resulted in defective aortic valve morphogenesis and/or septation of OFT. Nonetheless, to our knowledge, among all studies cited above, only three (23, 25, 28) extended their observations to the pathological changes of the AscA in connection to valve anomalies, without any particular reference to the state of aortic endothelium.

## EndMT/EMT, a Complex Biological Process Activated During Normal Physiology as Well as During Pathological Conditions

**Figure 1:**
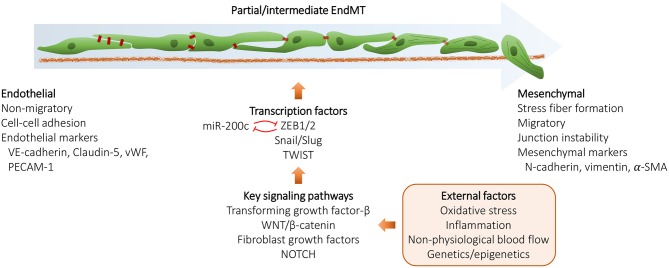
Schematic representation of the endothelial to mesenchymal transition (EndMT) process. During EndMT, endothelial cells lose endothelial cell features and acquire mesenchymal cell markers, resulting in junction instability, enhanced vascular permeability, and potentially cellular senescence. EndMT can be triggered by various external factors and involves signaling pathways, such as TGF-β, WNT/β-catenin, FGFs and NOTCH that converge and induce the expression of EndMT transcription factors ZEB1/2, Snail, Slug, and TWIST. EndMT/EMT may also be regulated by a number of microRNAs, the key microRNA family being miR-200 family that acts by suppressing ZEB1 and ZEB2 mRNA expression by a negative feedback loop.

Epithelial mesenchymal transition (EMT) is one of the most studied biological processes due to its fundamental role in the onset of cancer metastasis. EMT program induces epithelial cells to lose their adhesion, polarity and cell-cell junctions, restructure their cytoskeleton and become more invasive and motile i.e., acquire “mesenchymal” state (see Figure 1). However, EMT is also essential for normal physiological processes such as embryogenesis or wound healing, but may be aberrantly reactivated in pathological conditions (32, 33). EndMT, a specific form of EMT originally discovered in endocardium during cardiac development (34), shares many similarities with EMT and is activated during similar biological processes and diseases (35–37). EndMT/EMT can also be induced in response to certain environmental changes such as oxidative stress, inflammation, or hemodynamic changes (38). Several signaling pathways with major roles in embryogenesis such as, NOTCH, TGFβ, WNT, FGF, EGF, are also regulators of the EndMT/EMT program (33, 39). Although several transcription factors monitor EndMT/EMT, five of them, e.g., ZEB1/ZEB2, SNAI1/SNAI2, and TWIST are considered to be the key factors for activation of EndMT/EMT (40, 41).

Disruption of cell junctions is central to EndMT/EMT and that is achieved partly by transcription regulation of the junction proteins and partly by modification, turnover and degradation of junction proteins via endocytosis (42, 43). An early event in EndMT/EMT is the regulation of Cadherin superfamily expression, for instance downregulation of E-cadherin/VE-cadherin (*CDH1*/*CDH5*) in EMT and EndMT respectively, and upregulation of N-cadherin also known as mesenchymal cadherin (*CDH2*) (44, 45). Depending on the biological context and the outcome, EndMT/EMT is divided into three subtypes. Type I, which is activated during embryogenesis producing mesenchymal cells, Type II, occurring during tissue repair producing fibroblasts, and Type III, producing tumor cells activated during the metastatic propagation of cancers (33, 46) (Table 1).

**Table 1:** Occurrence and outcome of the different EndMT/EMT subtypes.

**Subtype**	**I**	**II**	**III**
**Biological context**	Embryogenesis	Tissue repair	Metastasis
**Cell type produced**	Mesenchymal cells	Fibroblasts	Tumor cells

Like many other biological processes, EndMT/EMT is regulated by a number of microRNAs (47). One of the key microRNA families governing this process is miR-200 family that acts by suppression of *ZEB1* and *ZEB2* mRNA expression by a negative feedback loop, thereby coordinating EndMT/EMT with Cadherin expression (48, 49).

## Altered Endothelial Function and Ascending Aortic Aneurysms: an End to SMC-only Dogma?

In spite of ample evidence presented for the role of endothelium in regulating the development, remodeling and functional integrity of vascular smooth muscle cells (VSMCs) (50–52), the aortic media has been in the center of attention in the aneurysm community. However, in late years, the involvement of the intima in aneurysm development has been brought into focus (53–55), which may be particularly interesting in the case of patients with BAV considering the numerous patient-based studies demonstrating exposure of non-physiological hemodynamic forces to the BAV AscA. One of the first experimental indications of endothelium involvement in aneurysm was the pioneering experiment with the Angiotensin II (ANGII)–infusion mice model where cell specific deletion of ANGII receptor, AT1a, in endothelial cells (EC) could attenuate ascending aortic aneurysm (56). Interestingly, the EC-specific transgenes showed structural reorganization of aortic media emblematic of aneurysm, arguing that the signals initiated in endothelium could induce aneurysm and accompanying pathological changes in the SMC. In a review article published 2013 (57), we discussed the intimal EC response to shear stress and the mechanisms by which this response could be relayed to and induce the pathological changes observed in media of AscAs in BAV aneurysmal patients. Furthermore, a recent and excellent review discusses the role of the endothelium and the potential mechanisms by which it influences the media layer in relation to the development of BAV-associated TAA, but with no focus on the EndMT/EMT process (58).

## Can Changes in Endothelial/Endocardial Functions that Give Rise to a BAV also Influence the Developmental Program of Ascending Aorta?

The crucial role of cells with endothelial/endocardium origin in the morphogenesis of aortic valve and OFT have been established by numerous experimental studies in transgenic animals (59–65) (Table 2).

**Table 2:** Selection of transgenic mice studies analyzing the effect of certain mutations on aortic valve formation and integrity.

**Mutation**	**Phenotype**	**References**
*Nos3*^−/−^	27% (15/55) of *Nos3^−/−^*embryos with BAV	(21)
*Nos3*^−/−^	40% (5/12) of mature *Nos3^−/−^*mice with BAV	(66)
*Gata5*^−/−^	25% (7/28) of *Gata5^−/−^*mice with BAV	(67)
Endothelial cell-specific *Gata5*^−/−^	21% (3/14) of mice with BAV	(67)
Endocardial lineage-specific *Brg1*^−/−^	Heart defects, semilunar valve malformations, BAV (3/6 at E16.5)	(68)
Endothelium-specific *Gata4*^−/−^	Diverse malformations affecting processes leading to valve formation	(69)
Deletion of *Vangl2* in SHF	Abnormal valve leaflets	(70)
*Robo1*^−/−^	Membranous ventricular septal defects (6/10 at E14.5)	(71)
*Robo1*^−/−^*;Robo1*^−/−^	Membranous ventricular septal defects (3/3 at E14.5), BAV (3/3 at E18.5)	(71)
*Slit2*^−/−^	Membranous ventricular septal defects (2/6 at E14.5), BAV (1/7 at E18.5)	(71)
*Slit3*^−/−^	Membranous ventricular septal defects (2/5 at E14.5)	(71)
*Robo4*^−/−^	Aortic valve defects (7/7), aortic aneurysm	(15)

*BAV, bicuspid aortic valve; SHF, second heart field*.

Several animal models with either a mutated endothelial specific gene or EC specific gene mutations were shown to develop progenies with BAVs. Mice deleted for endothelial specific nitric oxide synthase (eNOS the product of *NOS3* gene) gave rise to 40% progeny with a BAV (66). Laforest et al. (67) were the first to propose that aberrant EC migration and differentiation was associated with the formation of a BAV in *GATA5*^−/−^mice mutants with 25% BAV progeny. EC-specific *GATA5* deletion also resulted in mice progeny with a BAV, and *GATA5*^−/−^ offspring with BAV had significantly lower expression of endothelial specific markers, *CDH5* and *TIE2*, as well as decreased expression of eNOS in their left ventricles and OFT in comparison to the wildtype, indicative of altered endothelial function reminiscent of ROBO4 mutations in human BAV (see below). Unfortunately, neither of these studies addressed the extent or existence of concomitant aorthopathy or the status of AscA intima. Nonetheless, EC-specificity of NOS3 together with the fact that *GATA5* is restricted mostly to the endocardium, disappears at mid gestation stage, and is required for early differentiation of cardiac progenitors into endothelial/endocardial cells (72) hints to a connection between BAV phenotype and disturbed endothelial function.

The association between functional integrity of AscA endothelium, inheritance of a BAV, and development of aneurysm has now been experimentally demonstrated in a newly published report (15). In this study, the association of heterozygous mutation of *ROBO4*, a gene important for vascular integrity by regulating endothelial barrier function (73), with non-syndromic familial inheritance of human BAV was established. Histological examination of sections from AscA of a patient with heterozygote mutation of *ROBO4* and AscA aneurysm compared to control individual with matching age and sex demonstrated decreased intimal expression of *ROBO4*, increased fibro-proliferative phenotype of intima and sub-intimal region, and disrupted endothelial barrier function as judged by albumin staining. Treatment of human aortic ECs with siRNA against *ROBO4* or expression of a mutant variant in the presence of inhibited endogenous *ROBO4* resulted in loss of endothelial barrier function accompanied by downregulation of EC-adherence junction cadherin, *CDH5*, and tight junction component *TJP1* at mRNA and protein levels and transformation of these cells to a mesenchymal state. Moreover, a direct involvement of SLIT-ROBO signaling in the formation of cardiac cushion and inheritance of BAV has been shown (71), and expression of ROBO4 in the endothelium of the aortic valve and proximal AscA was shown to persist throughout the mice postnatal life (15). Loss of function mutations of *ROBO4* in mice revealed thickened aortic valves with or without BAV and in some cases, AscA aneurysm. These animals showed low penetrance and male predominance characteristic of human BAV (15). These observations nicely tied the inheritance of BAV and aneurysm of AscA to EC breakdown of barrier function and acquisition of mesenchymal state.

Changes in endothelial barrier function is most probably only limited to the proximal AscA in BAV patients and should have resulted from the impaired interaction between cardiac progenitors at the point where the common fate of aortic valve and AscA is determined. In line with that, using lineage tracing of specific markers of SHF mesenchyme showed that these progenitors could give rise to both SMC and endothelium of OFT (31, 64, 74) and the SHF specific markers could be traced both in the endocardium and endothelium of the developing AscA (75, 76), implying close developmental ties between these tissues in early embryogenesis. Indeed, it is thus likely that the impaired interaction also alters the VSMC population in the BAV aorta, rendering it more sensitive to TAA development.

## Impaired Mesenchymal Boundaries During Development and Its Consequences for Formation of BAV and Associated Aortopathy

During the development of aortic valves and OFT, there is a defined boundary for mesenchyme produced by endocardium, NCC and SHF progenitors (77). A defective mesenchyme production by each could be compensated for by the extension of other compartments into the segment that was not their normal niche. This change of mesenchymal boundaries and compensation by others has been shown to give rise to abnormal formation of aortic valves and septation of OFT (21, 31, 78, 79) as well as progenies with BAV (21, 68). Normally, the AscA is populated by a mosaic of SMCs arising from SHF mesoderm and migrating cardiac NCC, and recently, it was demonstrated in mice that NCC stemmed SMCs reside in areas close to intima and SHF generated SMC lie closer to the adventitia (76, 80). One possibility is thus that the impaired signal exchange between cardiac progenitors during development of aortic valves and OFT affects the distal OFT in such a way that a higher proportion of cardiac NCC populate the AscAs in BAV, filling some of the territory normally occupied by SMC of SHF origin.

One line of evidence supporting this hypothesis is the link between cancer cells and embryonic neural cells. Inhibition of a few chromatin modification enzymes in several cancer cell lines resulted in the loss of malignant features and differentiation to neuron-like cells. Further, a major part of mesenchymal marker genes activated during cancer promotion were only expressed in embryonic neural cells, including NCC, and not in other types of embryonic cells suggestive of a common regulatory network between tumorigenesis and neural development (81). If aortic media has a higher content of SMC with NCC origin in BAV, the appearance of EMT signals and cancer-related metabolic pathways among differentially regulated pathways between BAV and TAV is expected. In addition, the SMCs developed from NCC are more “immature” and proliferative and less contractile. As the elastin deposition in arteries takes place during fetal development, NCC-originated SMC may have different elastic properties. Whether or not a diversion from normal proportions of SMC could change the composition of collagen and elastic fibers is not known. All these factors can contribute to increased vulnerability of a cardinal vessel with high pressure function such as aorta.

## Endothelial Abnormality in BAV Patients

Although it is almost impossible to disentangle the genetic from hemodynamic causes, distortion of endothelial-related functions in aneurysmal tissue of BAV patients has been described in several patient-based studies. For example, circulating endothelial progenitor cells as a marker of EC repair efficiency were significantly lower in BAV compared to TAV patients (82) as well as being lower in BAV patients with aortic regurgitation or stenosis compared to functional BAVs (83). In a study of male subjects of comparable age, systemic endothelial dysfunction was reported in BAV patients with proximal aortic dilation compared to non-dilated individuals with a BAV (84). Using multivariate analysis comparing BAV and TAV patients with dilated and non-dilated AscA, BAV morphology turned out to be the main predictor of increased circulating PECAM^+^ endothelial-specific microparticles, independent of the type of cusp fusion or disease (31). Notably, circulating PECAM^+^ microparticles were significantly decreased in patients who underwent aortic valve surgery, establishing endothelial damage in BAV individuals probably due to exposure to non-physiological blood flow, although the possibility of an inherited defect synergizing hemodynamic factors cannot be ruled out. Several laboratories that focused on eNOS content in the AscA reported a differential expression of eNOS between BAV and TAV aneurysmal patients, both at transcriptional and translational levels (85–88).

In a recent study, we provided cytological evidence for intimal instability and induction of EndMT-like process in non-dilated AscA of BAV patients due to downregulation and enhanced protein turnover of VE-Cadherin (*CDH5*) in addition to decreased expression of endothelial specific Claudin-5 (*CLDN5*). Moreover, mRNA expression of N-cadherin (*CDH2*) increased in dilated AscA of BAV patients compared to dilated AscA of TAV patients (16). Further, we showed that alteration in cadherin expression was accompanied by formation of pseudopodia and stress fibers in endothelium of non-dilated BAV, which is a second key hallmark of transition to a mesenchymal state.

## Non-Physiological Shear Stress and Induction of EndMT/EMT

A pertinent issue to raise here is if the prenatal, as well as lifelong exposure of BAV AscA to non-physiological flow, could contribute to the induction of EndMT/EMT in AscA of adult individuals. Using different set-ups, the influence of shear stress on the induction of EndMT in EC have been studied (29, 89–92). During embryonic development, subjecting the OFT to increased hemodynamic load via banding was shown to enhance the EndMT of the cardiac cushion in chicken embryos (93, 94). Also, changes in blood flow by banding of thoracic aorta resulted in enhanced EndMT/EMT in regions exposed to disturbed flow in mice (29). Indeed, DNA methylation studies performed by us further supported the notion that EndMT/EMT induction may be partly due to the exposure of the AscA to disturbed flow. Also, we further observed that the methylation signature in non-dilated BAV aorta was significantly associated with a methylation profile associated with oscillatory flow. Further, several key EMT transcription factors, such as ZEB1, SNAI2 and TWIST1 became hypomethylated in EC subjected to oscillatory flow indicating their increased activity. In addition, BAV and TAV primary ECs showed a different response to perturbed flow, with substantially fewer genes changing their expression in BAV ECs, indicating an impaired flow-response of BAV ECs.

In prenatal life, the exposure of cardiovascular system to blood flow starts with the onset of the first heartbeat and the early valve primordia has been shown to perform functions that are equivalent to the mature valves of adult heart (95, 96). For instance, ablation of NCC in quail embryos resulted in malformation of the OFT endocardial cushion and valves with consequent disturbed hemodynamic in OFT (31). Hence, impaired EndMT during cushion formation causing the formation of abnormal semilunar valves, would simultaneously subject the primitive aorta to non-physiological hemodynamic stresses at early stages of cardiogenesis. As hemodynamic factors function hand in hand and in parallel to genetic factors from early stages of OFT morphogenesis, it is difficult to separate the relative importance of each one for the induction of EndMT/EMT in AscA.

## Cell Communication and SMC Phenotype

Proper cell-cell communication between different arterial layers is fundamental for vascular function and integrity. Intimal shear stress and intimal/medial strain will be propagated to other vascular layer, influencing structure and function. In response to shear stress, EC-SMC communication can influence SMC phenotype and proliferation (47, 50, 97–102) via mediators such as microRNAs (31, 81, 103), gap junction (104), or activation of certain signaling pathways through ligand receptor interaction (105–108). Another possible route of cell-cell communication is the inclusion of extra vesicular bodies by endocytosis that can transfer molecular characteristics between different cell types (109). Thus, a variety of molecular messengers are capable of transferring the EndMT/EMT induced in one section to other vascular compartments.

We have previously performed comparative studies on BAV and TAV aortic intima-media specimen, from non-dilated or dilated aortas, to investigate differences at genomic, proteomic and epigenomic levels. First, by combining large-scale proteomic pathway analysis on differentially-expressed proteins in non-dilated aortas, we showed enrichment of genes belonging to EMT, protein degradation and trafficking, cell junction dynamics, apoptosis, cell cycle and cancer-related biological processes (16). Second, to identify possible regulatory microRNAs (miRs) underlying the observed protein signature, we combined proteomic data with an *in-silico* network analysis approach (110). This procedure identified the miR-200 family, known to be important regulators of EndMT/EMT activity (48), as a key modulator of the ongoing biological process that differs between non-dilated BAV and TAV aorta. (48). Lastly, DNA methylation studies further showed enrichment of EMT genes in non-dilated AscA of BAV patients (111). Similarly, analysis of intima-media in dilated AscA of BAV and TAV patients identified EMT as the top GO term and several key transcription factors for EMT, including *ZEB1, SNAI2*, and *TWIST2*, were hypomethylated in dilated BAV aorta.

Collectively, these results, at the levels of mRNA expression, proteomic, DNA methylation, and microRNA regulation displayed an “EMT” signature in the aortic intima-media, and a major consideration would be what EndMT/EMT-like processes could mean for the state of medial SMC. One possible explanation is the different proliferative capacity of aortic SMC between BAV and TAV. Several upregulated proteins in non-dilated aorta of BAV compared to TAV patients included proteins associated with increased cell proliferation and invasion. One clear example was Yes-Associated Protein 1 (YAP1) that has been shown to regulate division and differentiation of VSMC from cardiovascular progenitors (112, 113), particularly in NCC-derived SMC (114). Furthermore, we documented significantly higher expression of Ki67 protein in SMC nuclei of non-dilated AscA of BAV patients (16). This result is in line with reported “immaturity” of SMC in dilated and non-dilated AscAs of BAV patients (14, 115). Compatible with that, an epigenetic study of dilated AscA of BAV patients, found a strong and significant non-CpG hypomethylation in aortic media that was interpreted as high proliferative SMC in this region (116).

## Does Repair Deficiency in BAV Ascending Aorta Lead to Aneurysm Susceptibility?

Repair deficiency may be due to genetically impaired production or recruitment of stem/progenitor cells, and/or inefficient induction of repair-promoting signaling pathways. In non-regenerating adult tissues such as the aorta, the existence of mechanisms that can instigate the terminally differentiated VSMCs to resume proliferative cycle is vital for vascular repair. In the past decade, reservoirs of different stem/progenitor cells that can migrate and prime the vascular repair have been discovered (117) and the importance of functional stem/progenitor cells, their number, and the maintained proliferative capacity in vessel homeostasis have been discussed. Several different sources of VSMC progenitors or stem cells that can dedifferentiate and participate in repair and remodeling in physiological or disease situations have been identified (118, 119). These cells either reside within the adult VSMC tissues or reach the damaged VSMC via circulation or migration from the neighboring tissues (117–119). Adventitia, particularly in the aorta, has long been recognized as a main niche for vascular progenitors. The source of progenitors within adventitia is situated in the border area between the outer media and inner adventitia within a region highly rich in sonic hedgehog (SHH) signaling (119, 120). Nonetheless, in several arteries including the aorta, a subset of adventitial stem cells produced by fully differentiated residential VSMC has been reported to migrate into the adventitia to enrich the adventitial pool of vascular stem cells (58). One possibility for the higher susceptibility of AscAs to aneurysm in individuals with a BAV is an impaired function of the adventitia. Unfortunately, adventitia in BAV has also been neglected by aneurysm researchers. Studying adventitia, particularly the SHH rich region may be relevant for high susceptibility to aneurysm in BAV patients.

Searching for markers of SMC immaturity, Roostalu et al. (119) identified CD146/MCAM that was expressed by a small sub-population of SMC in adult descending aortas at sub-intimal regions and around aortic branch points and bifurcations, which remained immature also in adult tissue. Interestingly, the expression and activation of YAP1 protein, an inhibitor of VSMC differentiation (112) and the regulator of CD146 transcription (121), was also higher in these regions. This SMC sub-population was shown to proliferate and perform arterial repair in the case of minor injuries while severe injuries were repaired by cells migrating from adventitia. Whether or not such a subset of VSMC exists in human vs. mouse aorta and is populating the branching points of coronary arteries or arteries stemming from AscA or aortic arch is not clear. Nonetheless, we see an upregulation of YAP1 protein in non-dilated AscA of BAV patients (16) and gene expression analyses (13) revealed an increase of CD146/MCAM mRNA in dilated vs. non-dilated BAV AscA raising the possibility of increased immaturity in VSMC in BAV resulted from dilation. A systematic study of the role and regional distribution of CD146 expression and its relationship to VSMC “immaturity” of AscA in BAV may further clarify underlying mechanism of aortic dilation in BAV aortas.

## Premature Aging and BAV Aortopathy

One of the prime consequences of high proliferation of SMC and repair deficiency is premature aging. In line with this, Grewal et al. proposed that susceptibility to aneurysm in BAV was due to the SMC immaturity while in TAV was due to inflammation and enhanced aging (14, 115). This interpretation was questioned by Forte and Della Corte who proposed “premature aging” instead of immaturity being the cause of aortopathy in BAV (122). In support of premature aging, significantly shorter telomere length and lower wound healing capacity of aneurysmal SMC isolated from BAV as compared to control donors and TAV was reported (123). The two interpretations may not be mutually exclusive and induction of EndMT/EMT in BAV aorta can reconcile and encompass both proposals.

Aging and senescence are not equivalent and while aging organisms accumulate senescent cells, senescence can function as a response to a variety of stress situations unrelated to aging. Telomere shortening was originally believed to be the major cause of cellular senescence. However, several other inducers of senescence independent of telomere shortening have lately been identified, including hypoxia, oncogene-induced senescence, exposure to UV or gamma radiation, loss of tumor suppressing genes and mitochondrial dysfunction (124–127). Cellular senescence induced by telomere shortening is an intrinsic part of cell cycle check point causing permanent growth arrest and endowing the organism with tumor suppressive activity. Relevantly, cellular senescence and EndMT/EMT have both been considered as biological mechanisms guiding cancer progression and metastasis and recent findings marks the discovery of a cross talk between the two processes (128, 129). An example of such a crosstalk in non-cancerous cells is the induction of EndMT described for aging human aortic EC (130). Moreover, activation of all key transcription factors of the EndMT/EMT process has been reported in senescence (128, 129). ZEB1 has particularly been shown to be the link between cellular senescence and EMT (50). Thus, the immaturity in BAV can turn into premature senescence with all accompanying consequences. Our collective data on differentially regulated pathways between BAV and TAV i.e., hypoxia, oncogene-related pathways such as WNT or MYC, and UV response may well be due to premature senescence (16, 110, 111), giving support to the possibility of EndMT/EMT conversion to senescence and aortic degeneration in BAV. Compatible with shorter telomeres and senescence-induced activation of cell cycle checkpoint, we also observed significantly increased protein expression of the cyclin-dependent kinase inhibitor P27 (the product of *CDKN1B* gene) in non-dilated BAV AscA (16). Exposure to disturbed flow is a factor that can turn immaturity to premature aging. Indeed, disturbed flow was shown to stimulate senescence in ECs in mouse models and cell culture by a P53/P21-dependent mechanism (131).

## Concluding Remarks

The underlying molecular mechanisms for the BAV-associated aneurysm susceptibility remain to be elucidated. BAV patients differ in the extent and onset of medial degeneration and some individuals with a BAV may never develop aneurysm. The notion of disturbed signal exchange between cardiac progenitors causing distorted mesenchymal boundaries in aortic walls has backing in transgene studies and provides a molecular framework to explain some of these ambiguities. An extensive population of less differentiated, less contractile SMCs with NCC origin in the sub-intimal area of the aorta, the dysfunctional intima, in addition to constant exposure to non-physiological blood flow, subject the walls to a high risk. The degree of aortic susceptibility may depend on the location of the boundary and the magnitude of NCC contribution. Several different genes mentioned above, and many more yet to be discovered, can influence the boundary determination in OFT that can explain the difficulties of identifying responsible factors in the inheritance of non-familial BAVs.

Another level of complexity that may influence the disease outcome is the EndMT/EMT program itself. Recently, the process of EndMT/EMT has been revisited and it is currently defined as a way to provide more “plasticity” to the tissue. To perform that task, many EndMT/EMT transcription factors also interact with or recruit epigenetic modifiers in addition to their direct role in the regulation of transcription (41, 132). Moreover, the notion of EndMT/EMT being a transition between two alternative states of “epithelial” and “mesenchymal” have been challenged and most studies point to the tissues remaining in an intermediate state harboring both epithelial and mesenchymal features (40, 41). This state is termed partial or intermediate EndMT/EMT or “metastable” and can even become a final state of the tissues in some disease cases (40, 41). In the case of cancer, the existence of some pre-cancerous stem cells maintaining some, but not all, of the genetic features of cancer cells, that can be complemented and produce a fully transformed cell upon epigenetic changes have been reported (132). To extrapolate these ideas to aneurysm, the non-dilated aorta in BAV may be in a “metastable” state of EndMT/EMT or using cancer terminology, in a pre-cancerous state that may stay stable or be shifted further to a more mesenchymal state and aortic dilation. This is supported by the more aggravated EMT signature in dilation of BAV reported by us. In addition, the extent of the shift could depend on a spectrum of events such as cellular context, different pathways and/or different transcription factors initiating the transition, degree of epigenetic modifications, environmental cues, individual genetic background, and a wide range of other factors that may influence the outcome of aortic diseases.

## Author Contributions

All authors listed have made a substantial, direct and intellectual contribution to the work, and approved it for publication.

### Conflict of Interest

The authors declare that the research was conducted in the absence of any commercial or financial relationships that could be construed as a potential conflict of interest.
